# Strength Analysis and Stress-Strain Deformation Behavior of 3 mol% Y-TZP and 21 wt.% Al_2_O_3_-3 mol% Y-TZP

**DOI:** 10.3390/ma14143903

**Published:** 2021-07-13

**Authors:** Liliya Vladislavova, Tomasz Smolorz, Nina Orlovskaya, Mykola Lugovy, Michael J. Reece, Stefan Kӧbel, Agnieszka Kopia, Malgorzata Makowska, Thomas Graule, Gurdial Blugan

**Affiliations:** 1 Laboratory for High Performance Ceramics, Empa, Swiss Federal Laboratories for Materials Science and Technology, 8600 Duebendorf, Switzerland; Liliya.Vladislavova@empa.ch (L.V.); Thomas.Graule@empa.ch (T.G.); Gurdial.Blugan@empa.ch (G.B.); 2 Faculty of Metals Engineering and Industrial Computer Science, AGH University of Science and Technology, 30-054 Krakow, Poland; Tomasz.Smolorz@empa.ch (T.S.); kopia@agh.edu.pl (A.K.); 3 Department of Mechanical and Aerospace Engineering, University of Central Florida, Orlando, FL 32816, USA; 4 Institute for Problems of Materials Science, 03142 Kyiv, Ukraine; ver@ipms.kiev.ua; 5 School of Engineering and Materials Science, Queen Mary University of London, London E1 4NS, UK; m.j.reece@qmul.ac.uk; 6 Decema GmbH, 78224 Singen, Germany; s.koebel@decema.de; 7 The Paul Scherrer Institute (PSI), 5232 Villigen, Switzerland; malgorzata.makowska@psi.ch

**Keywords:** zirconia, ferroelasticity, hysteresis, strength, fracture toughness

## Abstract

The mechanical behavior of 3 mol% Y_2_O_3_-ZrO_2_ ceramic and 21 wt.% Al_2_O_3_-3 mol% Y_2_O_3_-ZrO_2_ ceramic composite with submicron grain size was studied. Mechanical properties, such as hardness, Young’s modulus, four-point bending strength, and fracture toughness of both materials were measured. Linear stress-strain deformation behavior of both 3 mol% Y_2_O_3_-ZrO_2_ and 21 wt.% Al_2_O_3_-3 mol% Y_2_O_3_-ZrO_2_ was observed in flexure, both at room temperature and at 400 °C. A small deviation from linear elastic deformation was detected in 21 wt.% Al_2_O_3_-3 mol% Y_2_O_3_-ZrO_2_ ceramic composite when loaded above a stress of 1500 MPa. Therefore, it was concluded that only elastic deformation occurred at low stresses upon loading, which exclude the presence of domain switching in zirconia upon bending under the loading conditions of this study.

## 1. Introduction

The unique material with high mechanical properties, 3 mol% Y_2_O_3_-ZrO_2_ ceramic (Y-TZP) has been intensively studied in the past [1,2,3]. The interest in the mechanical properties of Y-TZP has been high for a long time, as this ceramic shows very high strength and sufficiently high fracture toughness, which allow it to be used in ceramic knives, dental and other biomedical implants, bearing, and wear applications among others [3,4,5,6,7,8,9]. Y-TZP has also caused a lot of fundamental interest due to the occurrence of stress-induced martensitic tetragonal to monoclinic phase transformation [1]. The *t*→*m* transformation occurs in the stress field of a loaded crack tip and is responsible for the strengthening and toughening of tetragonal ZrO_2_ as compared to cubic fully stabilized 8 mol% Y_2_O_3_-ZrO_2_ [3]. In addition to transformation toughening, ferroelastic domain switching is another toughening mechanism acting in ZrO_2_ because of the presence of different defects [10]. Y-TZP ceramics are heavily twinned, and a number of potentially mobile defects such as twins, antiphase boundaries, oxygen vacancies, and stacking faults could all contribute to the absorption of mechanical energy during ZrO_2_ loading leading to domain switching and further toughening. As a result of transformation toughening and domain switching, the reported bending strength of 3 mol% Y-TZP varies in the range of 650–1200 MPa, and its fracture toughness varies in the range of 5 to 10 MPa·m^1/2^ [11], while fully stabilized 8 mol% Y_2_O_3_-ZrO_2_ has strength in the range of 200–300 MPa and fracture toughness in the range of 1.8–2.4 MPa·m^1/2^ [10].

The addition of Al_2_O_3_ to Y-TZP ceramics to improve mechanical properties was explored in a number of publications [12,13,14,15]. A significant increase in strength of Al_2_O_3_-Y-TZP (ATZ) composites was reported in [13,14,15]. The three-point bending strength of 28 vol% Al_2_O_3_-3 mol% Y_2_O_3_/ZrO_2_ composite was reported to be equal to 2800 MPa in [13,14], and in four-point bending was reported to be equal to 2400 MPa in [15]. Both transformation toughening of the Y-TZP phase in the composite and the presence of residual thermal stresses appearing due to the mismatch of the coefficients of thermal expansions between Al_2_O_3_ and Y-TZP phases, along with a decrease in grain size of the material, are likely to be responsible for the remarkable increase in bending strength of ATZ even when compared to already strong Y-TZP ceramics [12]. The reported values of fracture toughness of 28 vol% Al_2_O_3_-3 mol% Y_2_O_3_/ZrO_2_ ceramics are equal to 4.7 ± 0.5 MPa·m^1/2^ in [12] and to ~5 MPa·m^1/2^ in [15]. An increase of K_1c_ of ATZ up to ~10 MPa·m^1/2^ was reported in [15], however, the content of Y_2_O_3_ additive in ZrO_2_ was not specified in this paper. In addition to such an improvement in strength, ATZ ceramics showed better wear resistance and decreased degradation behavior in the presence of moisture, thus allowing it to be used for many biomedical applications, such as hip and dental implants.

## 2. Stress-Strain Deformation Behavior of ZrO_2_-Based Ceramics

The presence of the *t*→*m* phase transition or domain switching as toughening mechanisms will be evident in the deformation behavior, where one should be able to detect their presence in the stress-strain deformation plots of ZrO_2_-based ceramics upon loading. However, many important publications on the mechanical behavior of numerous ZrO_2_-based ceramics describing *t*→*m* phase transformation [1,16,17], or domain switching [10] as toughening mechanisms acting in ZrO_2_ upon loading, do not provide stress-strain deformation diagrams, and it is not possible to see how the material deforms.

### 2.1. Effect of t→m Phase Transition

A schematic stress-strain diagram presented in [18] shows a characteristic deformation behavior, where the material first starts deforming elastically up to a certain critical stress level, where the strain continues to grow, but the stress remains at a constant value due to the *t*→*m* transformation. Only when the transformation ends does the stress start growing again, and the material starts deforming elastically again all the way to failure. While the schematic stress-strain diagram presented in [18] was used to perform theoretical calculations of transformation toughening in tetragonal ZrO_2_, very similar stress-strain diagrams were obtained experimentally using a combination of hydrostatic and uniaxial compression loading in [19]. In these diagrams, the initial elastically deformed phase of 12 mol% CeO_2_-ZrO_2_ (Ce-TZP) exhibited a much steeper slope all the way up to ~1300 MPa loading, where a significant increase in strain at the constant stress level occurred, followed by a further increase in stress with another linear portion of stress-strain deformation, this time, however, with significantly lower slope and, thus, lower Young’s modulus of the material. Texture in transformed monoclinic ZrO_2_ phase and non-transformed tetragonal ZrO_2_ phase 12 mol% CeO_2_-ZrO_2_ was detected as a result of the transformation. Such texture formation in the *t*-ZrO_2_ phase was explained either by slip/rotation to accommodate monoclinic variants or by domain switching [19]. The formation of microcracks was also reported to occur in Ce-TZP during the *t*→*m* phase transition [20]. It is considered that the *t*→*m* phase transition can occur only at the free surface of the material upon loading; therefore, this mechanism is most effective upon failure at the crack tip. However, *t*→*m* transition “plateau” during stress-strain deformation of Ce-TZP can occur because of microcracking that creates free surfaces inside of the material allowing for the appearance of the monoclinic phase with a larger volume as compared to the initial tetragonal phase leading to the absorption of energy and related enhancement of mechanical properties.

A slightly different deformation behavior was obtained when measuring the stress-strain diagrams of 8 mol% MgO-ZrO_2_ (Mg-PSZ) ceramics [15,19,21]. Both triaxial [19] and uniaxial [21] compression and four-point bending [15] stress-strain plots of 8 mol% MgO-ZrO_2_ showed first elastic deformation, but upon reaching some critical “yield” stress, non-linearity was introduced. The yield stress reported in [Chen, Lankford] by triaxial and uniaxial compression was measured to be in the range of 900–1200 MPa, and the deviation from linear deformation and introduction of plasticity in Mg-PSZ during bending experiments were observed around 300–600 MPa [15]. In all loading experiments of Mg-PSZ, no ”plateau” was observed in the stress-strain deformation diagrams. It is known that 12 mol% CeO_2_-ZrO_2_ consists of pure tetragonal phases upon sintering; however, 8 mol% MgO-ZrO_2_ consists of a cubic matrix phase with tetragonal precipitates homogeneously distributed in the bulk of the cubic matrix [19]. Therefore the mechanisms of the *t*→*m* phase transformation and texture formation might be different in these two materials resulting in the difference of their stress-strain deformation diagrams.

The stress-strain diagrams of 3 mol% Y_2_O_3_-ZrO_2_ ceramics in compression are presented in [22]. The elastic stress-strain behavior was established to occur up to a very high compressive stress, where the yield stress and deviation from linear elastic deformation are reported at an impressive ~2800 MPa and all the way until failure at ~3400 MPa. The flexural strength of this Y-TZP polycrystalline ceramic is reported as ~1000 MPa; however, no stress-strain flexure diagrams were provided [22]. It was explained that the continuous non-step nature of transformation strain observed in Y-TZP appeared because the activation of the *t*→*m* transition upon loading depends on the microstructure of the ceramic. The grains in this polycrystalline Y-TZP have different grain size, morphology, Y_2_O_3_ content, and orientation relative to the stress axis and, therefore, a different applied stress is required to cause *t*→*m* transformation in each of these different grains. As a result, no autocatalytic “plateau” appeared in the stress-strain deformation plots of Y-TZP ceramics upon loading in compression [22].

### 2.2. Effect of Domain Switching

In addition to the *t*→*m* transformation acting in tetragonal ZrO_2_, domain switching was also proposed as a different toughening mechanism in ferroelastic ZrO_2_ [10]. In ferroelastics, the stress-strain deformation behavior is characterized by the presence of hysteresis with spontaneous strain and coercive stress [23]. Domain switching in ferroelastic ZrO_2_ occurs by reorientation of the tetragonal lattice distortion by external mechanical stress. In order to accommodate lattice distortion, the tetragonal ZrO_2_ is heavily twinned. However, the presence of such domains is an insufficient condition for ZrO_2_ to exhibit ferroelasticity, as the existing domain walls must also have sufficient mobility to be able to move upon application of the stress. For polycrystalline ceramics, the coercive stress necessary to initiate domain wall movement must be different for different grains, depending on grain size and orientation, availability of porosity, etc. The stress-strain plots of Ce-TZP obtained in uniaxial compression [10] exhibited a linear elastic behavior all the way up to 1650 MPa, where the appearance of a “plateau” was recorded with increasing deformation at an almost constant stress. The behavior was very similar to the one reported in [19]; however, in this experiment, the Ce-TZP sample was not loaded till failure, but it was unloaded to zero stress with an observed hysteresis loop and remnant strain. In [24], the deviation from linear elastic deformation was also reported to occur at 1650 MPa in 3 mol% Y-TZP loaded in uniaxial compression, with the exception that no “plateau” was detected and only non-linear deformation was recorded to occur above 1650 MPa in compression. In [24], the compression tests were coupled with in-situ neutron diffraction experiments, and therefore the domain switching as the formation of texture and preferred orientation in tetragonal Y-TZP above 1650 MPa was found. It was concluded that no preferred orientation was found below 1650 MPa when Y-TZP deformed elastically, and the texture formation/domain switching was responsible for ferroelastic behavior of ZrO_2_ during loading above 1650 MPa in compression.

Unlike elastically deformed ceramics, ferroelastics can demonstrate asymmetric deformation behavior in tension and compression. As a result, in ferroelastics, coercive stresses measured in tension and compression can be different. The coercive stress in compression for 3 mol% Y-TZP was reported to be equal to 1650 MPa [24]; however, the coercive stress in tension for this material was reported to be in the range of 300–600 MPa, which is significantly lower than the stress measured in compression. However, to the best of our knowledge, no stress-strain diagrams of Y-TZP measured in tension are available in the published papers, unlike published stress-strain diagrams of Y-TZP measured in compression. Furthermore, the authors could not identify the previous work where the cyclic stress-strain deformation behavior was performed at 400 and 600 °C.

### 2.3. Al_2_O_3_-Y-TZP Composites

In Al_2_O_3_-Y-TZP composite ceramics, besides *t*→*m* transformation toughening and domain switching, one can expect at least one more strengthening mechanism to occur, such as residual thermal stresses, which will affect both the strength and fracture toughness of this particulate composite [Pezotti]. However, no stress-strain deformation diagrams of Al_2_O_3_-Y-TZP ceramics were found in the literature.

### 2.4. The Goal of the Research

Here we report stress-strain deformation diagrams for 3 mol% Y-TZP and Al_2_O_3_-3 mol% Y-TZP ceramics measured in four-point bending along with their other selected mechanical properties, as no stress-strain deformation behavior of those two materials measured in tension could be found published in the literature.

## 3. Experimental

Commercially available 3 mol% Y_2_O_3_-ZrO_2_ powder (TZ-3YS-E, Tososh, Tokyo, Japan) was used for producing 3 mol% Y-TZP ceramics. The powder was uniaxially compressed at 20 MPa using a steel die into disks with an 82 mm diameter and 10 mm height, and then cold isostatically pressed at 200 MPa. The disks were further sintered at 1550 °C for 4 h to become almost fully dense. The sintering at different temperatures did not produce such dense Y-TZP ceramics. Commercial ATZ samples were provided by Decema (ATZ20-HIP, Decema GmbH, Singen, Germany). A powder with 20 wt.% Al_2_O_3_-3Y-TZP composition was molded into 8 mm diameter and 80 mm height cylinders by a proprietary colloidal shaping process, which was followed by pressureless sintering and hot isostatic post compaction. Y-TZP ceramic samples were produced in the laboratory using commercially available powders, while ATZ ceramics were produced by the industrial partner.

After sintering, 3 × 4 × 48 mm^3^ ceramic bars were machined to be used in four-point bending tests to measure fracture toughness and flexure strength and to study stress-strain behavior. Out of the 30 total Y-TZP machined samples, 10 of them were machined following a procedure (machining #1) that resulted in relatively low surface roughness, while the other 20 bars were machined following a slightly different procedure (machining #2), which resulted in higher surface roughness. Out of the 20 total ATZ machined samples, 10 of them were machined following a procedure (#1), the same as Y-TZP ceramics, which also resulted in lower surface roughness of the ATZ bars, while 10 other bars were machined following procedure #2, which resulted in higher surface roughness of the ATZ samples after machining. The surface roughness of all samples after machining was measured by a surface profilometer (Hommel tester T500). Some of these ceramic samples were mirror polished to prepare them for hardness measurements, phase analysis and microstructural characterization.

The density of machined samples was measured by Archimedes’ technique [25]. The microstructure and fracture surfaces of the Y-TZP and ATZ ceramics were analyzed using scanning electron microscopy (VEGA TS3, TESCAN, Brno, Czech Republic). The V-notches placed in the samples for fracture toughness measurements were documented using optical microscopy (Light Microscope Stereo Discovery.V20, Zeiss, Jena, Germany). The micrographs of the polished and thermally etched ceramic surfaces were used to measure the Y-TZP and ATZ grain size according to [26]. The phase composition of Y-TZP and ATZ ceramics was studied using X-ray diffraction. X-ray powder diffraction studies were performed in Bragg–Brentano geometry using a BRUKER AXS D8 ADVANCE diffractometer equipped with Cu-Kα X-ray source. The data was acquired in 2θ range of 5–120° with a step of 0.02° and a 1 s exposure time per step. Hardness tests were performed using a Vickers hardness tester Durimet (Ernst—Leitz, Wetzlar, Germany). The 9.8 N load was applied to the polished surface of the samples using a diamond indenter for 15 s, with 20 impressions placed on each Y-TZP and ATZ sample. The impression diagonals were further measured to determine hardness values in accordance with the EN 843-4 standard. The impulse excitation technique (IET) was also employed to measure the Young’s modulus of Y-TZP and ATZ ceramics [27].

Four-point bending strength was measured using a 2 kN load cell in a 40/20 mm loading geometry and a crosshead displacement speed of 1 mm/min (USM, Haller, Zwick Z005, Germany) using displacement control mode loading [28]. Each sample was preloaded to 10 N. Thirty Y-TZP samples, and twenty ATZ samples were taken to failure, and the obtained strength data were further analyzed using standard Weibull statistics [29]. The parameters of the two-parameter Weibull distribution, the scale parameter (σ_0_) and the Weibull modulus (*m*) were calculated by the maximum likelihood method. As no deformation of the samples was measured using a specialized measurement system in these four-point bending tests, the strain was estimated, taking into account testing machine compliance using the methodology described in [30].

The fracture toughness of V notched Y-TZP and ATZ ceramics was measured using the single edge v-notch beam (SEVNB) technique according to [31]. In addition, the fracture toughness of the material can also be estimated from the strength test samples, as the critical stress intensity factor of the semielliptical surface crack of the fracture origin, when dimensions of fracture origin and fracture stress in bending $\sigma_{b}$ are known. If this is the case, the fracture toughness can be calculated using,

(1) $$K_{1c}=H\sigma_{b}\sqrt{\pi \frac{a}{Q}}F\left(\frac{a}{t},\frac{a}{c},\frac{c}{b},\phi \right)$$

where *a* is the depth of the surface crack, *c* is the half-length of the surface crack, *t* is the sample thickness, *b* is the half-width of the sample, $H=H\left(\frac{a}{t},\frac{a}{c},\phi \right)$ is the correction factor, $Q=Q\left(\frac{a}{c}\right)$ is the shape factor for an elliptical crack, and $F\left(\frac{a}{t},\frac{a}{c},\frac{c}{b},\phi \right)$ is the stress-intensity boundary-correction factor. The calculation of $K_{1c}$ and determination of H, Q, and F is described in detail in [32]. Equation (1) is valid for 0<a/c≤1, 0≤a/t<1, c/b<0.5 and 0≤ϕ≤π.

A separate set of four-point bending experiments in cyclic loading was also performed on Y-TZP and ATZ ceramics at room temperature, as well as at 400 and 600 °C, in order to detect any hysteretic behavior of the ceramics during loading. The 3 × 4 × 48 mm^3^ samples were loaded using a 2 kN load cell and a bending jig with 5 mm rollers and 40/20 mm loading geometry on a universal testing machine (Zwick/Roell Z005, Zwick, Ulm, Germany). The tests were performed in a load control mode with a loading/unloading rate of 4 N/s. Each sample was preloaded to 10 N, which was set as a zero point for further loading. In cyclic bending experiments, three rod-deflection measurement systems (Maytech, Wuppertal, Germany) were used to measure the displacement of the samples. The deflection of the samples was measured by the central rod of the deflectometer positioned at the center of the tensile surface of the sample with two control rods positioned below the loading rollers at a distance of 10 mm from each side of the central rod. Therefore, during the cyclic loading experiments, both the applied load and deflection of the samples were directly recorded during the experiments. Once the samples were preloaded, and a zero load was set up, a cycling loading with 100 MPa increment was applied. The cyclic loading was applied all the way to a predetermined stress, which was done to ensure that failure did not occur during cycling. Therefore, Y-TZP experienced eight loading cycles all the way to 800 MPa, and ATZ experienced nine loading cycles all the way to 900 MPa at RT. However, Y-TZP tested in cycling loading at 600 °C failed during the fifth cycle, therefore for all elevated temperature tests at 400 and 600 °C, only four cycles were performed both for Y-TZP and ATZ ceramics.

## 4. Results and Discussion

### 4.1. Density, Grain Size, and Phase Composition of Y-TZP and ATZ

**Figure 1:**
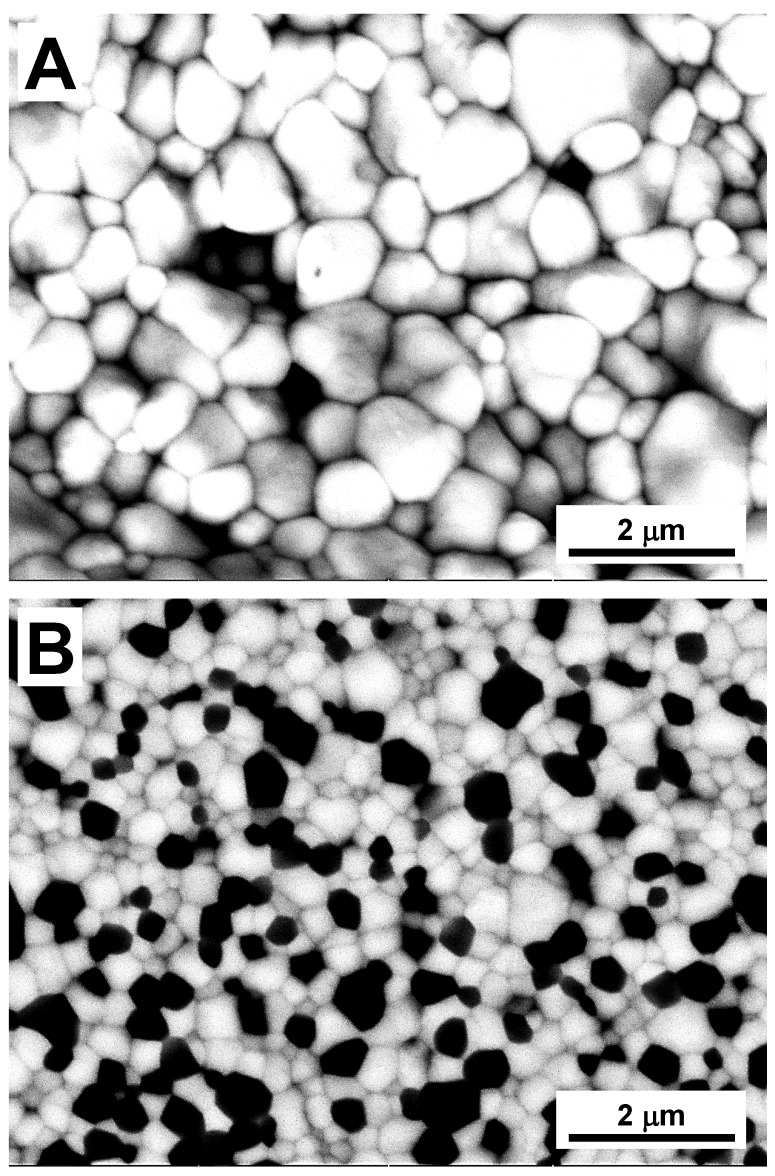
Backscattered SEM micrographs of (**A**) Y-TZP and (**B**) ATZ ceramics.

After sintering, the density of Y-TZP and ATZ ceramics were measured to be equal to 6.05 and 5.48 g/cm^3^, respectively (Table 1). Such high densities were indicative that both materials were sintered to almost full density. Only very small amounts of porosity were present in both ceramics. The microstructure of Y-TZP and ATZ ceramics is presented in Figure 1, and both Y-TZP and ATZ ceramics have a submicron grain size. The average grain size of Y-TZP ceramics was measured to be 0.735 ± 0.021 μm, while the ATZ ceramic composite has a much smaller average grain size of 0.414 ± 0.023 μm (Table 1). While no separate measurements were performed to calculate the grain sizes of the Al_2_O_3_ and Y-ZrO_2_ grains in the ATZ composite, one can see from Figure 1B that both phases have approximately similar grain sizes. By analyzing the phase composition of the Y-TZP ceramics, it was determined that the tetragonal phase was dominant at approximately 87 wt.%, while the cubic phase was also present.

**Figure 2:**
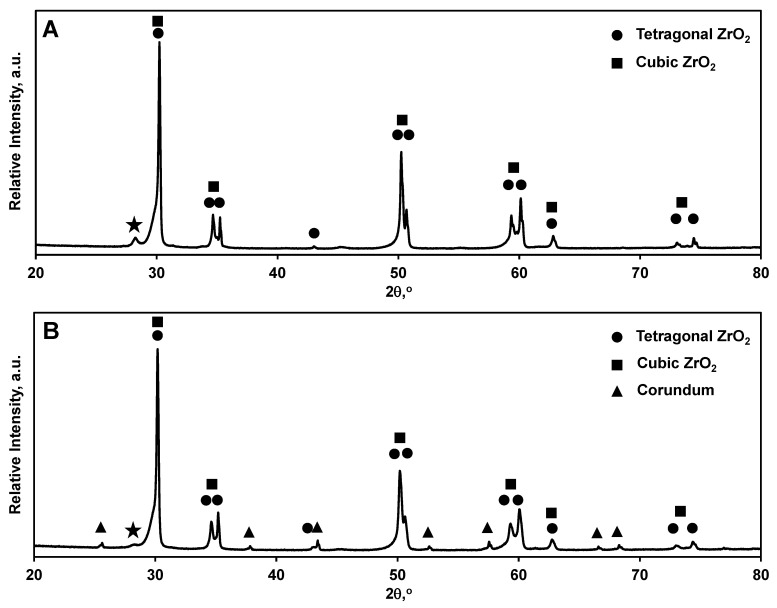
X-ray diffraction patterns of (**A**) Y-TZP and (**B**) ATZ ceramics. The small quantity of monoclinic ZrO_2_ phase identified by ★ on both diffraction patterns.

Where $X_{c}$ is the cubic ZrO_2_ phase fraction, $I{\left(400\right)}_{c}$ is the integrated intensity of cubic (400) peak, and $I{\left(400\right)}_{t}$ and $I{\left(004\right)}_{t}$ is the integrated intensity of tetragonal (400) and (004) peaks, respectively. A very small quantity of monoclinic ZrO_2_ can also be seen from the diffraction pattern (Figure 2); however, its presence might be explained by *t*→*m* phase transformation that occurred during machining of ZrO_2_ samples in preparation for mechanical testing. From the diffraction pattern of ATZ, one can identify the expected α-Al_2_O_3_ phase and both the tetragonal and cubic structures of the ZrO_2_ phase. Similar to Y-TZP, the ZrO_2_ consisted of 87% tetragonal phase and about 13% cubic phase, with the t to c phase ratio being very similar to that determined in pure Y-TZP ceramics. A small quantity of m-ZrO_2_ along with a small amount of a non-identified phase were also detected. No Rietveld refinement and no calculation of lattice parameters of the phases were performed.

### 4.2. Hardness and Young’s Modulus

The Vickers hardness of Y-TZP ceramics was measured to be equal to 12.08 ± 0.21 GPa (Table 2). The measured values correspond very well to the hardness of 3 mol% Y-TZP ceramics published in the literature, where Vickers hardness is reported in the range of 10–13 GPa [35,36]. The addition of a harder phase, such as Al_2_O_3_, increased the hardness of the composite, the hardness of the ATZ composite was measured to be equal to 13.91 ± 0.24 GPa (Table 2). As the hardness of pure Al_2_O_3_ was reported to be equal to ~19 GPa [37], the hardness of 20 wt.% Al_2_O_3_-Y-TZP ceramic composite could be estimated by a rule of mixture. It was estimated that the theoretical hardness value of the ATZ composite is equal to 13.7 GPa, which corresponds very well with the experimentally measured values.

Young’s modulus of Y-TZP, as measured by the impulse excitation technique, was equal to 211 ± 0.57 GPa (Table 2), which corresponds very well with the results published in [38]. The Al_2_O_3_ ceramic has a higher Young’s modulus (~400 GPa [39]) when compared to Y-TZP; therefore, the Young’s modulus of the ATZ composite was measured to be higher as compared to Y-TZP and was equal to 254 ± 0.19 GPa (Table 2). The theoretical estimates of the ATZ composite’s Young’s modulus calculated by the rule of mixtures provide a value of 265 GPa, which is found to be in good agreement with the measured Young’s modulus values by the IET technique.

### 4.3. Flexural Strength and Weibull Statistics

**Figure 3:**
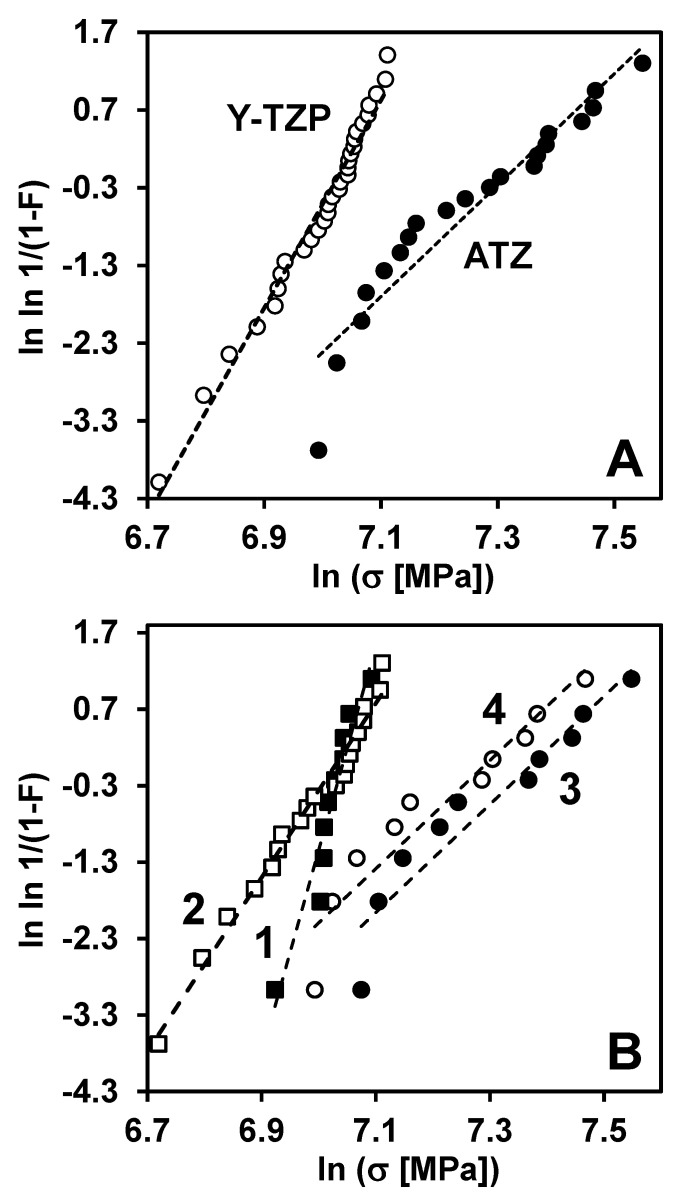
Weibull distribution of four-point bending strength of Y-TZP and ATZ ceramics. (**A**) Weibull plots, where all 30 samples of Y-TZP and 20 samples of ATZ ceramics were counted, (**B**) Weibull plots, where strength data of 10 samples of Y-TZP machined by a process #1 and 20 samples of Y-TZP machined by a process #2, along with strength data of 10 samples of ATZ machined by a process #1 and 10 samples of ATZ machined by a process #2 are shown separately for each composition with different machining.

The measured flexural strength of Y-TZP and, especially, ATZ was relatively high for ceramics. The average strength of Y-TZP ceramic was equal to 1106 ± 111 MPa, and the average strength of ATZ ceramic composite was equal to 1503 ± 229 MPa (Table 2). The Weibull distribution plots of all 30 tested Y-TZP, and all 20 tested ATZ samples are shown in Figure 3A. The experimental data, presented in Figure 3 are (*x*, *y*) pairs, where *x* = ln *σ*_b_ is a logarithm of the bending strength and $y=\ln\ln{\left(1-P_{f}\right)}^{-1}$ with $P_{f}$ being a fracture probability and the theoretical Weibull distribution, presented by the straight dashed line, which is the fit from the maximum likelihood function [29]. As one can see from Figure 3A, the experimental data obey the Weibull distribution almost perfectly for Y-TZP ceramics. However, deviations from the straight line were obtained for ATZ ceramics. The calculated Weibull parameters, the scale parameter (σ_0_) and the Weibull modulus (*m*) are presented in Table 3, where the scale parameters of Y-TZP and ATZ ceramics were equal to 1139 MPa and 1536 MPa, respectively, and Weibull moduli of Y-TZP and ATZ ceramics were equal to 13.4 and 7.2, respectively.

From the calculated Weibull moduli data, one can see that the lower deviations of experimental data of Y-TZP ceramics from the straight line in the Weibull plot may be accompanied by the higher Weibull modulus as compared to Weibull modulus of ATZ ceramics where significant deviations are observed. The scale parameter, which is representative of a “mean” strength, should be less affected by the deviations. However, the Weibull modulus, which characterizes uniformity of the flaw size distribution and distribution of the strength values, is very sensitive to the deviations. It is well known that the presence of deviations from the straight line in a Weibull plot can be explained by intrinsic differences in the critical defect size distributions, edge effects, or transition from volume to surface defects caused by machining. The R-curve behavior of ceramics can also contribute to the Weibull parameters, such as an increase in both the scale parameter by increasing the strength of the material and Weibull modulus by decreasing the sensitivity of the fracture to the non-uniform defects’ size distribution.

It was also of interest to analyze the obtained strength distribution of Y-TZP and ATZ ceramics based on the different machining procedures, which resulted in different surface roughness of these materials. Out of total of 30 samples of Y-TZP ceramics tested in four-point bending, 10 samples were machined following a proprietary procedure #1, which resulted in 0.1 μm surface roughness, and 20 samples were machined followed proprietary procedure #2, which resulted in 0.58 μm surface roughness. Out of the total 20 samples of ATZ ceramics, 10 samples were machined followed proprietary procedure #1, which resulted in 0.08 μm surface roughness, while the other 10 samples were machined followed proprietary procedure #2, which resulted in 0.56 μm surface roughness. These surface roughness results are presented in Table 1. The corresponding Weibull plots of all four different subsets of Y-TZP and ATZ ceramics of the samples machined by different procedures are shown in Figure 3B. As one can see from Figure 3B, the machining procedure of Y-TZP ceramics tremendously affected the Weibull modulus, as the uniformity of critical defects improved significantly with the decreasing surface roughness of the material. A surface roughness of 0.1 μm resulted in a Weibull modulus of 26.7 for Y-TZP, while for a surface roughness of 0.58 μm, the Weibull modulus decreased to 11.4. However, the scale parameter of Y-TZP ceramics was not so sensitive to the machining procedure, resulting in only the small deviation from 1145 to 1132 MPa when the machining resulted in the surface roughness of 0.1 μm and 0.58 μm, respectively. The different machining, resulting in different surface roughness, also affected the Weibull parameters of ATZ ceramics too, but in a different way compared to Y-TZP (Figure 3B). While the Weibull modulus showed no dependence and remained the same for the samples machined by different procedures, the scale parameter showed a higher value of 1599 MPa for the samples machined to a finer surface roughness of 0.08 μm, when compared to the scale parameter of 1474 MPa determined for the samples machined to the coarser surface roughness of 0.56 μm. Such results, the type of machining and different surface roughness do not significantly affect the uniformity of flow size distribution and distribution of strength values, indicate a better damage tolerance of ATZ ceramic composite compared to Y-TZP ceramics. However, to further investigate the different behavior, the fractography of the fracture surfaces of these materials was performed.

### 4.4. Fracture Surface Analysis and Sensitivity of Weibull Parameters to Critical Defect Size Distribution

**Figure 4:**
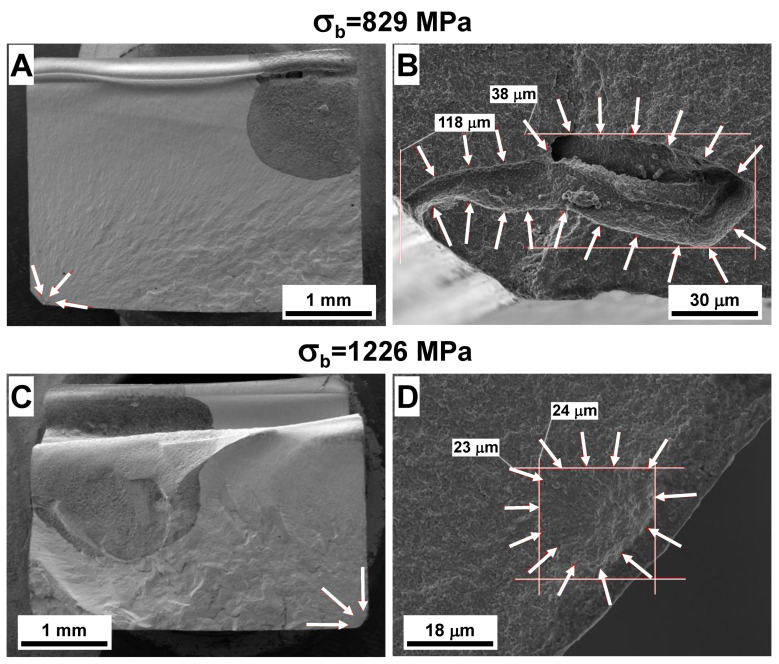
SEM micrographs of fracture surfaces of Y-TZP ceramics. The overall fracture surfaces of Y-TZP samples failed at 829 MPa (**A**) and 1226 MPa (**C**), where locations of fracture origins are marked with arrows. The fracture origins of Y-TZP samples failed at 829 MPa (**B**) and 1226 MPa (**D**). The dimensions of fracture origins in both cases are also shown.

**Figure 5:**
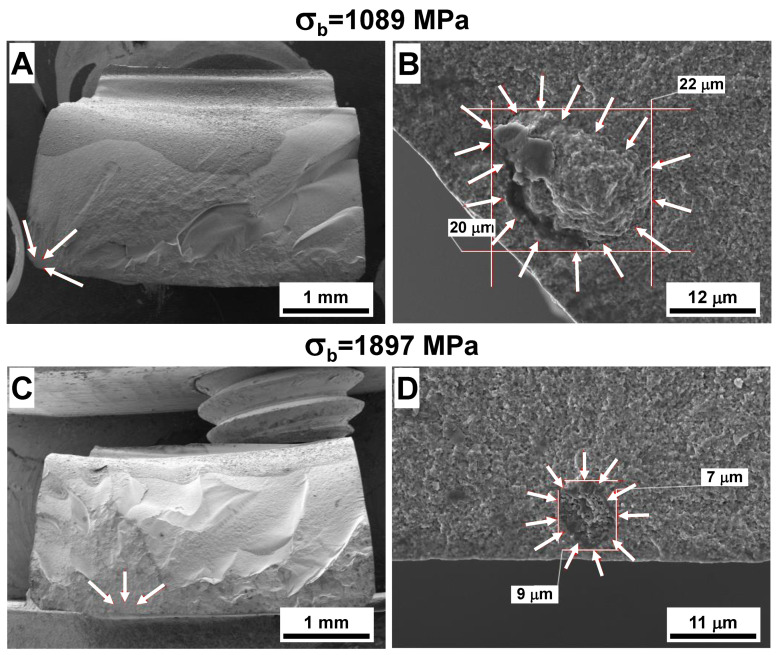
SEM micrographs of fracture surfaces of ATZ ceramic composite. The overall fracture surfaces of ATZ samples failed at 1089 MPa (**A**) and 1897 MPa (**C**), where locations of fracture origins are marked with arrows. The fracture origins of ATZ samples failed at 1089 MPa (**B**) and 1897 MPa (**D**). The dimensions of fracture origins in both cases are also shown.

SEM micrographs of the fracture surfaces of Y-TZP and ATZ ceramics after four-point bending are shown in Figure 4 and Figure 5, respectively. In both materials, the fracture surfaces of the samples with the lowest and highest strength values were analyzed. The SEM micrographs of the Y-TZP samples that failed at 829 and 1226 MPa are shown in Figure 4, the values represent the lowest, and the highest strength of Y-TZP measured on all 30 samples tested in four-point bending. The samples with the lowest and highest strength were machined using proprietary procedure #2, with a higher surface roughness of 0.58 μm after machining. The whole fracture surfaces of the two Y-TZP samples are shown in Figure 4A,C, from which the areas with a fracture origin were further identified. The magnified micrographs of larger defects, which served as fracture origins in Y-TZP ceramics with the lowest and highest strength, are shown in Figure 4B,D. As one can see from Figure 4B, the large defect that caused a fracture of the Y-TZP sample at 829 MPa was a large void, which was either produced due to different shrinkage of different agglomerates or some contamination that burned out during sintering, leaving this large defect inside the material. This defect, which served as the fracture origin where the crack started growing, causing the catastrophic failure, had the rather large dimensions of 38 × 118 μm^2^ and was also connected to the surface of the sample; thus it could be classified as a surface defect. The defect, which caused the failure of Y-TZP at 1226 MPa, was most likely also a relatively large subsurface pore with the dimensions of 23 × 24 μm^2^. This pore size was significantly smaller when compared with the previous sample, thus leading to a significantly larger stress the Y-TZP could withstand until it failed at 1226 MPa.

The SEM micrographs of the fracture surfaces of ATZ ceramics that failed at 1089 and 1897 Mpa are shown in Figure 5. Similar to Y-TZP, the fracture surfaces of ATZ samples failed at the lowest (1089 Mpa), and highest (1897 MPa) stress values were selected, as these values represent the lowest and the highest strength of ATZ measured from all 20 samples tested in four-point bending. The sample with the lowest strength of 1089 MPa was machined with proprietary procedure #2 and had a surface roughness of 0.56 μm after machining. The sample with the highest strength of 1897 MPa was machined with proprietary procedure #1 and had a surface roughness of 0.08 μm after machining. The whole fracture surfaces of these ATZ two samples are shown in Figure 5A,C. The magnified micrographs of larger defects, which served as fracture origins in the ATZ ceramic composite with the lowest and highest strength values, are shown in Figure 5B,D. The large defect that caused a failure of the ATZ sample at 1089 MPa appeared to be a large agglomerate with 20 × 22 μm^2^ dimensions, which differentially shrinks during the sintering of ATZ, leaving a quite big crack/void on one side. The defect which caused the failure of ATZ at 1897 MPa had rather small 7 × 9 μm^2^ dimensions leading to the ability of this sample to survive the loading to a relatively high stress of almost 1.9 GPa. In any case, the critical defect sizes of ATZ ceramic composite were significantly smaller when compared to Y-TZP ceramics, which explains the much higher strength of ATZ.

It is known that the Weibull distribution of strength data is directly related to the size distribution of critical defects that could serve as fracture origins in ceramics at failure. In two-parameter Weibull statistics, the average size of the critical defect determines a scale parameter, and a standard deviation of critical defect size distribution determines the Weibull modulus, where the large average size of critical defects will lead to a decrease in a scale parameter and the large standard deviation of critical defects’ size distribution will lead to a decrease in the Weibull modulus. The machining of ceramics will affect the size distribution of critical defects; therefore, it will affect the Weibull distribution of the strength data of Y-TZP and ATZ ceramics. In the case of Y-TZP ceramics, the critical defects have a larger average size but a lower standard deviation of size distribution, leading to a lower scale parameter and larger Weibull modulus when compared to ATZ ceramics. Such dependencies are determined by the fact that defects present in Y-TZP have larger dimensions in comparison to those defects present in the ATZ ceramic composite. This difference in the size of critical defects present in Y-TZP and ATZ ceramics has to be taken into account when looking for the explanation as to why different machining resulting in different surface roughness leads to different Weibull distributions in these two materials. The machining #1 with a final 0.1 μm surface roughness in Y-TZP most likely removes the coarsest defects resulting in a smaller average size and smaller standard deviation of critical defect size distribution when compared to the machining #2, which produces a surface roughness of 0.58 μm. Such removal of the largest defects during machining leads to an increase in both the scale parameter of 1145 MPa and Weibull modulus of 26.7 for samples after machining #1, when compared with the scale parameter of 1132 MPa and Weibull modulus of 11.4 for the machining #2 where surface roughness is much coarser and larger defects are present, leading to a premature failure of this ceramic.

However, the critical defects with such large sizes present in Y-TZP do not exist in the ATZ ceramic composite, where the average defect size should be smaller, allowing ATZ to achieve a very high flexural strength value of almost 1.9 GPa. Therefore, both machining #1 and machining #2 most likely remove defects more homogeneously in ATZ, simply because larger defects are not present in this ceramic. Machining #1 with a surface roughness of 0.08 μm results in a slight decrease in the average defect size in comparison with machining #2 with a surface roughness of 0.56 μm while the standard deviation of critical defect size distribution remains practically the same for both types of machining. Such an absence of large critical defects leads to the situation of a much lower sensitivity of Weibull parameters to the critical defect size distribution of ATZ ceramic composites, as the scale parameter increases slightly to 1599 MPa and Weibull modulus of 7.1 remains the same after machining #1 in comparison with the scale parameter of 1474 MPa and Weibull modulus of 7.1 after machining #2.

### 4.5. Fracture Toughness

**Figure 6:**
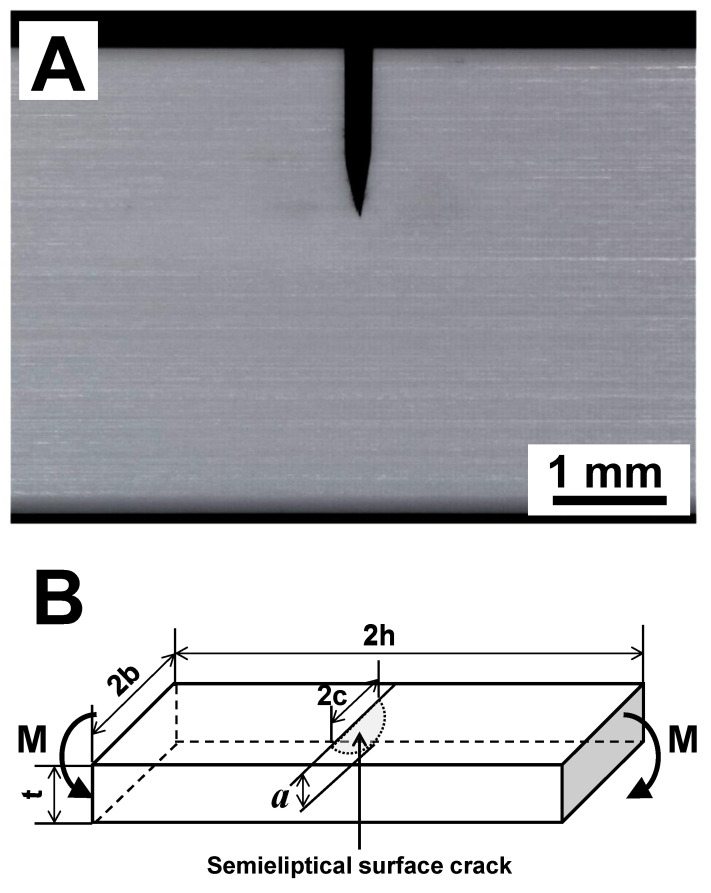
Critical defects in ceramics that are used for the calculation of fracture toughness. (**A**) A photo of a V-notch cut placed in the ceramic sample on the surface under applied tensile stress for SEVNB tests, (**B**) a schematic presentation of a semielliptical surface crack used in *K*_1*c*_ calculation proposed in [32].

The fracture toughness (*K*_1*c*_) of both Y-TZP and ATZ ceramics measured by the SEVNB method was equal to 5.38 ± 0.09 and 5.02 ± 0.41 MPa·m^1/2^, respectively (Table 2). A photograph of the V-notch placed in the Y-TZP is shown in Figure 6A, where the V-notch served as an artificial defect in the material, ensuring that the crack responsible for the catastrophic failure would necessarily originate from the notch tip upon loading. The measured *K*_1*c*_ values of Y-TZP correspond very well with data published in [11]; however, there are *K*_1*c*_ values of Y-TZP reported in the literature as high as 10 MPa·m^1/2^ [15]. The *K*_1*c*_ of ATZ ceramic composite measured in this work also corresponds very well with the data published in [13,14].

Other than the SEVNB method of measuring *K*_1*c*_ of Y-TZP and ATZ ceramics, there is another highly applicable technique that also allows an estimate of the fracture toughness of brittle materials [32]. In this technique, the origin of brittle failure is considered to be a critical defect originally present in the material, unlike an artificial defect introduced by notching of ceramics in the SEVNB technique. This critical defect is modeled as a semielliptical surface crack located at the tensile surface of the sample upon loading in bending (Figure 6B). When both dimensions of semielliptical surface crack and critical fracture stress are known, the fracture toughness of ceramic can be calculated using Equation (1). The estimations of the sizes of semielliptical surface cracks were performed based on the micrographs of the fracture origins of Y-TZP and ATZ ceramics with minimum and maximum strength values (Figure 4 and Figure 5). The sizes of the semielliptical surface cracks were estimated as *a* = 38, 2*c* = 94 μm and *a* = 23, 2*c* = 46 μm for minimum and maximum strength Y-TZP samples, and as *a* = 20, 2*c* = 40 μm and *a* = 7, 2*c* = 14 μm for minimum and maximum ATZ samples. Using these estimations, the calculated fracture toughness of Y-TZP samples with a strength of 829 and 1226 MPa was equal to 6.6 and 7.6 MPa·m^1/2^, respectively, while the calculated fracture toughness of ATZ samples with a strength of 1089 and 1897 MPa was equal to 6.3 and 6.5 MPa·m^1/2^, respectively. The difference in fracture toughness of Y-TZP and ATZ ceramics obtained using these two independent methods can be explained by the fact that when the notch is placed in the material for SEVNB testing, such a defect could be considered as a macrocrack. However, when a second technique is used for the calculation of fracture toughness, critical defects responsible for initiation of failure are relatively small and can be treated as microcracks. Furthermore, in this second technique, an approximation of the complex shape of the critical defects is performed using the semielliptical surface crack approach, which gives the ability to perform such calculations, however leading to the overestimates of *K*_1*c*_ values when compared to the SEVNB technique.

### 4.6. Stress-Strain Deformation Behavior in Bending

**Figure 7:**
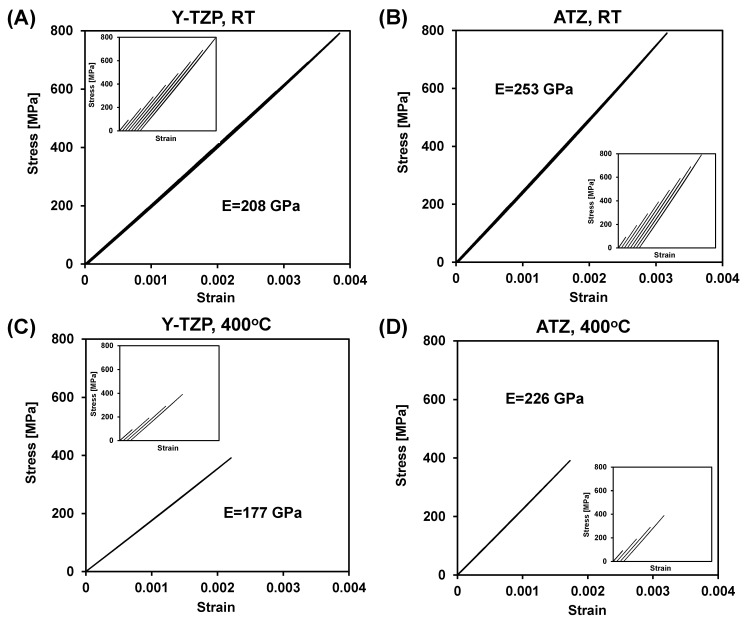
Cyclic stress-strain deformation plots of Y-TZP (**A**,**C**) and ATZ (**B**,**D**) ceramics measured at RT (**A**,**B**) and 400 °C (**C**,**D**).

The stress-strain deformation behavior of Y-TZP and ATZ ceramics measured at room temperature and at 400 °C is shown in Figure 7. Both materials were subjected to a room temperature cyclic loading. Eight loading/unloading cycles were performed with an incremental loading, subjected to a 100 MPa incremental stress increase between cycles (Figure 7A,B). The high-temperature cyclic loading of Y-TZP ceramics was performed at 600 °C first, where a sample failed at 447 MPa during the cycle upon loading. Therefore, in order to avoid failure, the cyclic testing at 400 °C of both Y-TZP and ATZ ceramics was done to a maximum stress of 400 MPa (Figure 7C,D). Each cycle of these tests is separately presented in the inserts shown in Figure 7. From the slopes of stress-strain diagrams, it was determined that the RT Young’s modulus of ATZ ceramic composite is indeed higher (254 GPa) when compared to Y-TZP ceramics (211 GPa), which is in good correspondence with the Young’s modulus measurements obtained by the impulse excitation technique. The Young’s moduli of both Y-TZP and ATZ ceramics decreased to 177 and 226 GPa, respectively, when the temperature of the cyclic flexure experiments was increased to 400 °C, which corresponds very well to similar results reported in [11,12].

The linear stress-strain deformation behavior of both Y-TZP and ATZ ceramics shown in Figure 7 is in contradiction with the appearance domain switching under tensile stress at room temperature, reported to occur at 300–400 MPa in 3 mol% Y-TZP upon bending [40]. Absolutely no deviation from linear elastic deformation in Y-TZP and ATZ was found in the current experiment at 300–400 MPa, both at RT and at 400 °C testings. While domain mobility was detected by in situ XRD in 3 mol% Y-TZP at stress ~400 MPa as reported in [40], no experimentally measured stress-strain deformation plot was provided for that experiment. Therefore it is not possible to make a direct comparison of stress-strain deformation behavior reported in the current paper and in [40].

**Figure 8:**
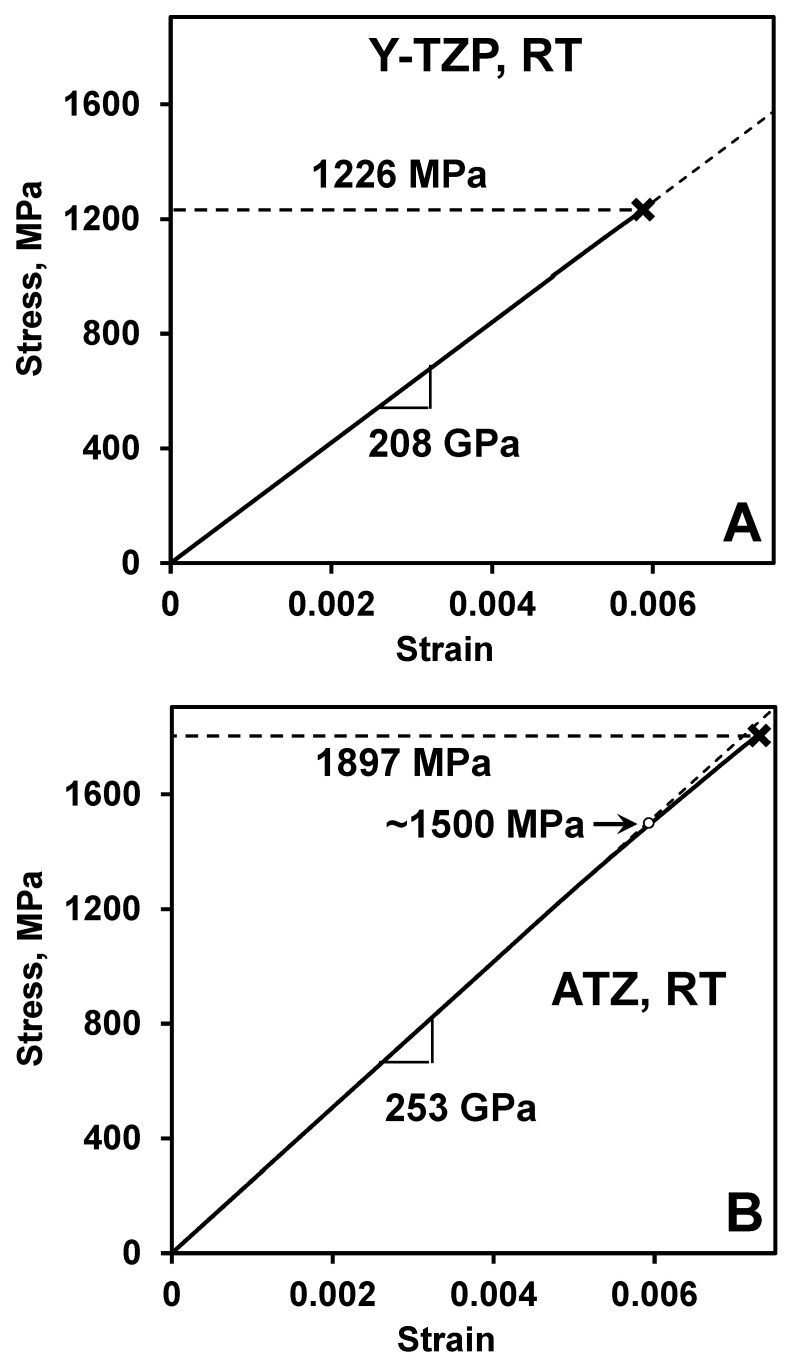
Stress-strain diagrams of Y-TZP (**A**) and ATZ (**B**), where a strain was reconstructed from a crosshead displacement and compliance of a universal testing machine using known Young’s moduli of the ceramics under loading.

Only linear elastic deformation was detected in Y-TZP and ATZ ceramics upon bending. However, the loading was performed to a maximum stress of 800 MPa at RT in order to avoid the failure of the ceramics during cycling. It would be important to determine what the deformation would be at the higher stress level during the four-point bending of Y-TZP and ATZ ceramics. We tested both of these materials, which failed at 1226 and 1897 MPa for Y-TZP and ATZ ceramics, respectively. However, during these tests, the deflection of the ceramic bar was not directly measured upon loading. Therefore, in such case, no direct strain calculation could be made. However, we used a technique proposed in [30], where the deflection of the sample can be extracted from the displacement of the crosshead of the universal testing machine measured during loading. As the Young’s moduli of Y-TZP and ATZ are known, it was possible to calculate the strain on the tensile surface of samples, which appeared during loading of the ceramics in four-point bending [30]. The stress-strain deformation curves of Y-TZP and ATZ ceramics, where the strain was estimated by the technique proposed in [30], are shown in Figure 8. The Y-TZP ceramic sample failed at 1226 MPa, and only linear elastic behavior was detected upon loading all the way until failure. However, the ATZ ceramic sample failed at 1897 MPa and, for this material, a slight deviation from linearity was detected, which began at ~1500 MPa.

It was determined in [24] that upon compression of 3 mol% Y-TZP, the domains become mobile, and texture starts to appear at rather high stresses above 1650 MPa. Their experiments confirmed that the domain switching occurred in the bulk of the material, as they performed in situ neutron-diffraction experiments on Y-TZP upon loading in compression. Therefore, the domains become mobile in ZrO_2_ at a relatively high-stress level, while at stresses below 1200 MPa, Y-TZP is linearly elastic and does not show any signs of ferroelastic hysteretic deformation. One of the possible explanations as to why it is so hard to move the domain walls in Y-TZP might be the fact that the Young’s modulus of ZrO_2_ is relatively high (210 GPa), while most ferroelastic ceramics have a much lower stiffness [41,42,43,44,45]. As the Young’s modulus is a characteristic of the bond strength in the material, a higher Young’s modulus is indicative that higher stresses are required to break bonds to allow domains to move upon loading. Therefore, while less stiff ceramics might have coercive stress of ~70 or ~125 MPa, the reported values, where a deviation from linearity occur in ZrO_2_, are much higher, as ZrO_2_ requires 1.5–1.6 GPa in order to exhibit either deviation from linearity or appearance of a plateau on the stress-strain deformation curves [24]. Such higher stresses required for ZrO_2_ to exhibit non-linearity could be caused by strong bonding and high Young’s modulus, as compared to other classical ferroelastic materials.

## 5. Conclusions

The mechanical properties of 3 mol% Y_2_O_3_ stabilized ZrO_2_ (Y-TZP) ceramics, and 21 wt.% Al_2_O_3_-Y-TZP (ATZ) particulate ceramic composite was studied with a particular emphasis on the stress-strain deformation behavior of these materials under flexure. Such mechanical properties as hardness, Young’s modulus, fracture toughness, and four-point bending strength were all measured to obtain a better understanding of the instantaneous mechanical behavior of Y-TZP and ATZ ceramics. Both the measured hardness and Young’s modulus values of these materials correspond very well with the results published in the literature. The fracture toughness measured by SEVNB on both materials is reported to be in the lower range of the published data. The *K*_1*c*_ evaluated using fracture origin as a critical defect where failure initiated showed slightly higher values compared to the *K*_1*c*_ values measured by the SEVNB technique. The flexural strength of both Y-TZP and ATZ ceramics was measured as very high, which is indicative that very large defects were removed and were not present significantly in the materials after processing. This is especially true for ATZ ceramic composite showing significantly smaller critical defects present in the material leading to the outstanding four-point bending strength. The probability of failure and reliability of these two materials were analyzed using two-parameter Weibull statistics. The cyclic loading was also performed during four-point bending experiments on Y-TZP and ATZ ceramics in an attempt to detect any sign of non-linearity and hysteresis. It was established that both Y-TZP and ATZ deform exclusively in a linear elastic way in cycling with no sign of hysteresis or any deviation from linearity both at room temperature and at 400 °C. The linear elastic deformation of Y-TZP and ATZ persists to rather high-stress values, and it is only at 1500 MPa and above that deviation from linear elastic behavior was detected in ATZ at room temperature. Y-TZP ceramics have shown a lower strength of ~1200 MPa at RT; therefore its deformation cannot be measured at 1500 MPa and above and, thus, no conclusion can be made about whether it would exhibit deviation from linear elastic deformation at a higher stress level on a tensile side upon bending experiments. Therefore, one can conclude that at the lower than 1500 MPa bending stress, there is no indication of domain switching occurring in Y-TZP and ATZ during bend loading. The deviation from linear deformation detected in the ATZ ceramic composite above 1500 MPa might be attributed to domain switching, and such high stress might require the cause of domain switching because of the very small grain size of this ceramic. The results correspond very well with published data, where domain switching in 3 mol% Y-TZP was detected by in situ neutron diffraction during uniaxial compression at the compressive stress above 1650 MPa.

## Figures and Tables

**Table 1:** Microstructural parameters of yttria-stabilized zirconia (Y-TZP) and alumina-zirconia composite (ATZ).

Property	Y-TZP	ATZ
Density, g/cm^3^	6.05	5.48
Porosity, %	0.8	0.04
Grain size, μm	0.735 ± 0.021	0.414 ± 0.023
Tetragonal phase fraction of *t*-ZrO_2_, %	87	87
Roughness Ra (machining #1), μm	0.1	0.08
Roughness Ra (machining #2), μm	0.58	0.56
$X_{c}=\frac{I{\left(400\right)}_{c}}{I{\left(400\right)}_{c}+I{\left(400\right)}_{t}+I{\left(004\right)}_{t}}$		[33,34]

**Table 2:** Mechanical properties of yttria-stabilized zirconia (Y-TZP) and alumina-zirconia composite (ATZ) at room temperature.

Property	Y-TZP	ATZ
Hardness, GPa	12.08 ± 0.21	13.91± 0.24
Young modulus (IET), GPa	211 ± 0.57	254 ± 0.19
Flexural strength, MPa	1106 ± 111	1503 ± 229
Fracture toughness, MPa·m^1/2^ (SEVNB)	5.38 ± 0.09	5.02 ± 0.41
Fracture toughness, MPa·m^1/2^ (estimated from fracture origin dimensions)	7.1	6.4
Young modulus (slope), GPa	208	253

**Table 3:** Weibull parameters of yttria-stabilized zirconia (Y-TZP) and alumina-zirconia composite (ATZ).

Composition	*m* (mixed)	σ_0_ (mixed), MPa	*m* (machin. 1)	σ_0_ (machin. 1), MPa	*m* (machin. 2)	σ_0_ (machin. 2), MPa
Y-TZP	13.4	1139	26.7	1145	11.4	1132
ATZ	7.2	1536	7.1	1599	7.1	1474

## Data Availability

The data presented in this study are available on request from the corresponding author.

## References

[1] Garvie R.C., Hannink R.H., Pascoe R.T. (1975). Ceramic Steel. Nature.

[2] Kelly J.R., Denry I. (2008). Stabilized zirconia as a structural ceramic: An overview. Dent. Mater..

[3] Chevalier J., Gremillard L., Virkar A.V., Clarke D.R. (2009). The Tetragonal-Monoclinic Transformation in Zirconia: Lessons Learned and Future Trends. J. Am. Ceram. Soc..

[4] Soubelet C.G., Albano M.P. (2019). Mechanical properties and aging behaviour of Y-TZP with 64S bioglass additions for dental restorations. Adv. Appl. Ceram..

[5] Zou J., Zhong Y., Eriksson M., Liu L., Shen Z. (2018). Tougher zirconia nanoceramics with less yttria. Adv. Appl. Ceram..

[6] Gjurin S.Z., Özcan M., Oblak C. (2019). Zirconia ceramic fixed partial dentures after cyclic fatigue tests and clinical evaluation: A systematic review. Adv. Appl. Ceram..

[7] Han J., Zhao J., Shen Z. (2016). Zirconia ceramics in metal-free implant dentistry. Adv. Appl. Ceram..

[8] Mondal B., Kundu S. (2006). Novel synthesis of advanced composites of α-Al_2_O_3_ reinforced with Ce TZP through co-precipitation process. Adv. Appl. Ceram..

[9] Li D., Liu Y., Zhong Y., Liu L., Adolfsson E., Shen Z. (2019). Dense and strong ZrO_2_ ceramics fully densified in <15 min. Adv. Appl. Ceram..

[10] Virkar A.V., Matsumo R.L.K. (1986). Ferroelastic domain switching as a toughening mechanism in tetragonal zirconia. J. Am. Ceram. Soc..

[11] Ruiz L., Readey M.J. (1996). Effect of heat treatment on grain size, phase assemblage, and mechanical properties of 3 mol% Y-TZP. J. Am. Ceram. Soc..

[12] Basu B., Vleugels J., Van Der Biest O. (2004). ZrO_2_–Al_2_O_3_ composites with tailored toughness. J. Alloy. Compd..

[13] Pezzotti G., Sergo V., Sbaizero O., Muraki N., Meriani S., Nishida T. (1999). Strengthening contribution arising from residual stresses in Al_2_O_3_/ZrO_2_ composites: A piezo-Spectroscopy investigation. J. Eur. Ceram. Soc..

[14] Shikata R., Urata Y., Shiono T., Nishikawa T. (1991). Mechanical properties and characterization of ZrO_2_-Al_2_O_3_ composites with high fracture strength. J. Jpn. Soc. Powder Powder Met..

[15] Swain M., Rose L.R.F. (1986). Strength Limitations of Transformation-Toughened Zirconia Alloys. J. Am. Ceram. Soc..

[16] Hannink R.H.J., Kelly P.M., Muddle B.C. (2004). Transformation Toughening in Zirconia-Containing Ceramics. J. Am. Ceram. Soc..

[17] Zeng Z., Liu Y., Zhang Y., Zhou Z., Liu X. (2020). Ferroelastic domain switching toughening in Ce–Y–La co-stabilized zirconia ceramics obtained from coated starting powders. J. Alloy. Compd..

[18] Mcmeeking R.M., Evans A. (1982). Mechanics of Transformation-Toughening in Brittle Materials. J. Am. Ceram. Soc..

[19] Chen I.-W., Reyes-Morel P.E. (1986). Transformation Plasticity and Transformation Toughening in Mg-PSZ and Ce-TZP. Symposium E—Advances in Structural Ceramics.

[20] Chen I.-W., Morel P.E.R. (1986). Implications of Transformation Plasticity in ZrO_2_-Containing Ceramics: I, Shear and Dilatation Effects. J. Am. Ceram. Soc..

[21] Lankford J. (1983). Plastic Deformation of Partially Stabilized Zirconia. J. Am. Ceram. Soc..

[22] Lankford J., Page R.A., Rabenberg L. (1988). Deformation mechanisms in yttria-stabilized zirconia. J. Mater. Sci..

[23] Orlovskaya N., Gogotsi Y., Reece M., Cheng B., Gibson I. (2002). Ferroelasticity and hysteresis in LaCoO_3_ based perovskites. Acta Mater..

[24] Cain M., Bennington S.M., Lewis M.H., Hull S., Cain M., Bennington S.M., Lewis M.H., Hull S. (1994). Study of the ferroelastic transformation in zirconia by neutron diffraction. Philos. Mag. B.

[25] Spierings A., Schneider M.J., Eggenberger R. (2011). Comparison of density measurement techniques for additive manufactured metallic parts. Rapid Prototyp. J..

[26] Mendelson M.I. (1969). Average Grain Size in Polycrystalline Ceramics. J. Am. Ceram. Soc..

[27] EN 843-2 (2006). Advanced Technical Ceramics—Mechanical Properties of Monolithic Ceramics at Room Temperature. Part 2: Deter-Mination of Young’s Modulus, Shear Modulus and Poisson’s Ratio.

[28] EN 843-1 (2006). Advanced Technical Ceramics—Mechanical Properties of Monolithic Ceramics at Room Temperature—Part 1: Determination of Flexural Strength.

[29] Orlovskaja N., Peterlik H., Marczewski M., Kromp K. (1997). The validity of Weibull estimators-experimental verification. J. Mater. Sci..

[30] Kalidindi S., Abusafieh A., El-Danaf E. (1997). Accurate characterization of machine compliance for simple compression testing. Exp. Mech..

[31] (2006). Advanced Technical Ceramics—Test Methods for Determination of Fracture Toughness of Monolithic Ceramics. Part 5. Single-Edge V-Notch Beam (SEVNB) Method.

[32] Newman J., Raju I. (1981). An empirical stress-intensity factor equation for the surface crack. Eng. Fract. Mech..

[33] Ghatee M., Shariat M.H., Irvine J.T.S. (2009). Investigation of electrical and mechanical properties of 3YSZ/8YSZ composite electrolytes. Solid State Ion..

[34] Paterson A., Stevens R. (1986). Phase analysis of sintered yttria–zirconia ceramics by x-ray diffraction. J. Mater. Res..

[35] Lin J.-D., Duh J.-G. (2003). Fracture toughness and hardness of ceria- and yttria-doped tetragonal zirconia ceramics. Mater. Chem. Phys..

[36] Gupta N., Mallik P., Basu B. (2004). Y-TZP ceramics with optimized toughness: New results. J. Alloy. Compd..

[37] Kaya C. (2003). Al_2_O_3_–Y-TZP/Al_2_O_3_ functionally graded composites of tubular shape from nano-sols using double-step electrophoretic deposition. J. Eur. Ceram. Soc..

[38] Ho C.J., Liu H.C., Tuan W.H. (2009). Effect of abrasive grinding on the strength of Y-TZP. J. Eur. Ceram. Soc..

[39] Sivanesan S., Loong T.H., Namasivayam S.N., Fouladi M.H. (2019). Two-Stage Sintering of Alumina-Y-TZP (Al_2_O_3_/Y-TZP) Composites. Key Eng. Mater..

[40] Jue J.F., Virkar A.V. (1990). Fabrication, Microstructural Characterization, and Mechanical Properties of Polycrystalline t′-Zirconia. J. Am. Ceram. Soc..

[41] Wiik K., Fossdal A., Sagdahl L., Lein H.L., Menon M., Faaland S., Wærnhus I., Orlovskaya N., Einarsrud M.-A., Grande T. (2004). LaFeO_3_ and LaCoO_3_ Based Perovskites: Preparation and Properties of Dense Oxygen Permeable Membranes. Mixed Ionic Electronic Conducting Perovskites for Advanced Energy Systems.

[42] Vullum P.E., Holmestad R., Lein H.L., Mastin J., Einarsrud M.-A., Grande T. (2007). Monoclinic Ferroelastic Domains in LaCoO_3_-Based Perovskites. Adv. Mater..

[43] Lein H.L., Andersen Ø.S., Vullum P.E., Lara-Curzio E., Holmestad R., Einarsrud M.A., Grande T. (2006). Mechanical properties of mixed conducting La_0.5_Sr_0.5_Fe_1−*x*_Co_*x*_O_3−*δ*_ (0 ≤ *x* ≤ 1) materials. J. Solid State Electrochem..

[44] Martin A., Khansur N.H., Webber K.G. (2018). Electric field-induced changes in the ferroelastic behavior of (Na_1/2_Bi_1/2_) TiO_3_-BaTiO_3_. J. Eur. Ceram. Soc..

[45] Webber K., Aulbach E., Key T., Marsilius M., Granzow T., Rödel J., Webber K., Aulbach E., Key T., Marsilius M. (2009). Temperature-dependent ferroelastic switching of soft lead zirconate titanate. Acta Mater..

